# Tissue engineering of human hair follicles using a biomimetic developmental approach

**DOI:** 10.1038/s41467-018-07579-y

**Published:** 2018-12-13

**Authors:** Hasan Erbil Abaci, Abigail Coffman, Yanne Doucet, James Chen, Joanna Jacków, Etienne Wang, Zongyou Guo, Jung U. Shin, Colin A. Jahoda, Angela M. Christiano

**Affiliations:** 1 Department of Dermatology, Columbia University Medical Center, New York, NY, 10032 USA; 2 Department of Systems Biology, Columbia University Medical Center, New York, NY, 10032 USA; 3 Department of Biosciences, Durham University, Durham, UK; 4 Department of Genetics and Development, Columbia University Medical Center, New York, NY, 10032 USA

## Abstract

Human skin constructs (HSCs) have the potential to provide an effective therapy for patients with significant skin injuries and to enable human-relevant drug screening for skin diseases; however, the incorporation of engineered skin appendages, such as hair follicles (HFs), into HSCs remains a major challenge. Here, we demonstrate a biomimetic approach for generation of human HFs within HSCs by recapitulating the physiological 3D organization of cells in the HF microenvironment using 3D-printed molds. Overexpression of *Lef-1* in dermal papilla cells (DPC) restores the intact DPC transcriptional signature and significantly enhances the efficiency of HF differentiation in HSCs. Furthermore, vascularization of hair-bearing HSCs prior to engraftment allows for efficient human hair growth in immunodeficient mice. The ability to regenerate an entire HF from cultured human cells will have a transformative impact on the medical management of different types of alopecia, as well as chronic wounds, which represent major unmet medical needs.

## Abstract (web summary)

Human skin constructs hold potential for regenerative medicine, but the incorporation of hair follicles into such constructs is a challenge. Here, the authors use 3D printed molds to pattern hair follicle cell types in a physiological organization, and achieve human hair growth on the back of a mouse.

## Introduction

Skin is a complex organ that contains more than 50 different cell types that comprise the core epidermal, dermal, and hypodermal tissues, as well as various other components, including vasculature, sensory neurons, the skin immune system, and appendages, such as hair follicles (HFs). Each year, more than six million patients are hospitalized in the U.S. for significant skin loss or disfigurement due to thermal and pressure injuries, chronic diabetic ulcers or genetic blistering skin diseases^1^. The ability to generate bioengineered human skin constructs (HSCs) has provided a promising skin replacement therapy for these patients^2^ and allowed for human-relevant drug screening to target skin disorders^3^.

Currently available HSCs still have significant limitations including poor long-term viability and lack of appendages, such as HFs, which play roles in thermoregulation, barrier function, and wound healing^4^. Our group recently improved the viability of skin grafts by establishing a method to micropattern induced pluripotent stem cell (iPSC)-derived vasculature in HSCs^5^. However, the incorporation of HFs into engineered HSCs remains a major challenge and limits their potential for regenerative medicine and preclinical drug testing.

Dermal papilla cells (DPCs) are highly specialized mesenchymal cells that are indispensable for HF morphogenesis and cycling. Previous studies have shown proof-of-concept for inducing human hair growth in mice through intracutaneous transplantation of intact DPCs and epithelial cells^6^. However, transforming this concept into a feasible therapeutic strategy requires large numbers of human DPCs, which raises a number of challenges due to the paradoxical rapid loss of hair inductivity of DPCs when expanded in 2D in vitro culture^7,8^. Various approaches have been used to restore the inductive characteristics of DPCs, such as co-culture with keratinocytes^9^, use of small molecules^8^, and hypoxia culture^10^. We previously showed that 3D-spheroid culture of DPCs could partially restore their intact transcriptional signature^7^. This method allowed us and other groups^11^ to induce human hair formation using cultured DPC spheroids in mice, albeit inefficiently due to high levels of variability in the hair inductive properties of DPCs.

Using a systems biology approach, we also identified several master regulator (MR) genes of inductive DPC identity, which could potentially be used to achieve complete restoration of hair inductive transcriptional signature of DPCs^7^. Similarly, we recently reported that Jak inhibitors also directly restore hair inductivity in treated DPCs in culture^12^. Following these substantial steps towards restoring intact DPC identity, we postulated that generation of de novo HFs in HSCs requires both significant reprogramming of the DPC transcriptional signature, as well as accurate recapitulation of critical microenvironmental cues, such as epithelial−mesenchymal and cell− extracellular matrix interactions.

In this study, we present an innovative biomimetic approach for effective generation of human HFs within HSCs by recapitulating the physiological 3D conformation of cells in the HF microenvironment. We exploit the unique capability of 3D-printing technology to create structures with high aspect ratios (length to width ratio: ~100 for human HFs^13^), which was not possible with previous microfabrication techniques, such as soft lithography. Our approach permits controllable self-aggregating spheroid formation of DPCs in a physiologically relevant extracellular matrix and initiation of epidermal−mesenchymal interactions, which results in HF formation in HSCs in vitro. Further, vascularization of hair-follicle-bearing HSCs increases graft survival and enables efficient human hair growth in mice. Our method represents a novel bioengineering strategy for feasible generation of hair-bearing HSCs entirely ex vivo from cultured human cells.

## Results

### Controlling 3D spatial arrangement of cells in HSCs

Human DPC spheroids have the potential to induce de novo hair formation when placed in contact with human epidermis in mice^7,11^. However, when spheroids were placed in HSCs and maintained in culture, they rapidly dissociated into the collagen matrix over several days and did not initiate the epidermal−mesenchymal interactions required for HF morphogenesis (Supplementary Figure 1). In agreement with previous studies^14^, this observation indicated that simple engineered HSCs do not provide a physiological microenvironment sufficient to induce HF formation.

**Fig. 1:**
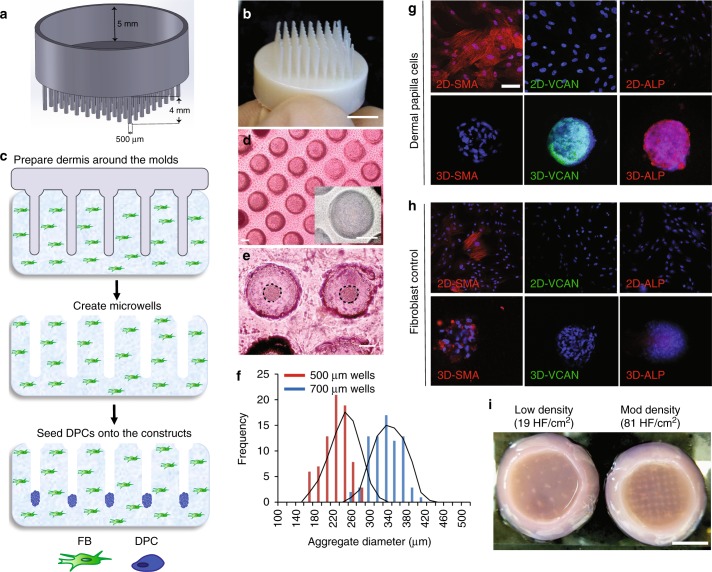
Patterning of collagen type-1 gel using 3D-printed molds allows for physiological arrangement of cells in the hair follicle. Hair follicle molds were designed (**a**) and 3D-printed (**b**) to have HF-like extensions and a 5-mm-deep cavity that allows the molds to float on collagen gel. **c** The collagen gel containing dermal fibroblasts was allowed to solidify around the HF-like extensions to create an array of microwells in which the DPCs formed spontaneous aggregates. **d** Top view of HSCs containing DPCs which settled down into the microwells (lower panel: higher magnification of the microwells). **e** DPCs formed spontaneous aggregates at the center of the microwells (dashed line circles the aggregates). **f** The size of DPC aggregates, as calculated from images, was correlated with the diameter of the microwells (500 µm wells in red and 700 µm wells in blue) (*n* = 9; three technical and three biological replicates). Expression of SMA and VCAN and the activity of ALP in 2D cultures of DPCs and in DPCs aggregates formed in the microwells (**g**) was compared to 2D cultures of FBs and FB aggregates in the microwells (**h**). **i** Top view of the 3D-reconstructed dermis at two different hair follicle densities of 19 HF per cm^2^ and 81 HF per cm^2^. Scale bars for (**d**) and (**e**−**h**) are 100 µm and 5 mm for (**b**) and (**i**)

To control the spatial arrangement of cells in HSCs, we used 3D-printing technology and microfabricated plastic molds that contain HF-shaped extensions that are adjustable in diameter, length, and density (Fig. 1a, b). These molds were used to create an array of microwells on a type I collagen gel containing dermal fibroblasts (FBs) (Fig. 1c). Seeding DPCs over these microwells led to spontaneous aggregate formation in the base of the microwells overnight (Fig. 1d, e). This method allowed for precise control of DPC aggregate size by adjusting the diameter of the microwells (Fig. 1f). The spontaneous aggregate formation restored the expression of versican (VCAN) and alkaline phosphatase (ALP) activity and suppressed the expression of smooth muscle actin (SMA), a protein only expressed in 2D cultures of DPCs but not in intact DP in vivo (Fig. 1g). These changes were not observed in FB aggregates (Fig. 1h), which we used as a negative control throughout the study. The 3D-printing approach allowed us to generate HSCs with different HF densities, such as 19 and 81 HF per cm^2^ (Fig. 1i), and to achieve an in vivo-like DPC phenotype in 3D-reconstructed dermis.

### Induction of hair follicle differentiation in HSCs

**Fig. 2:**
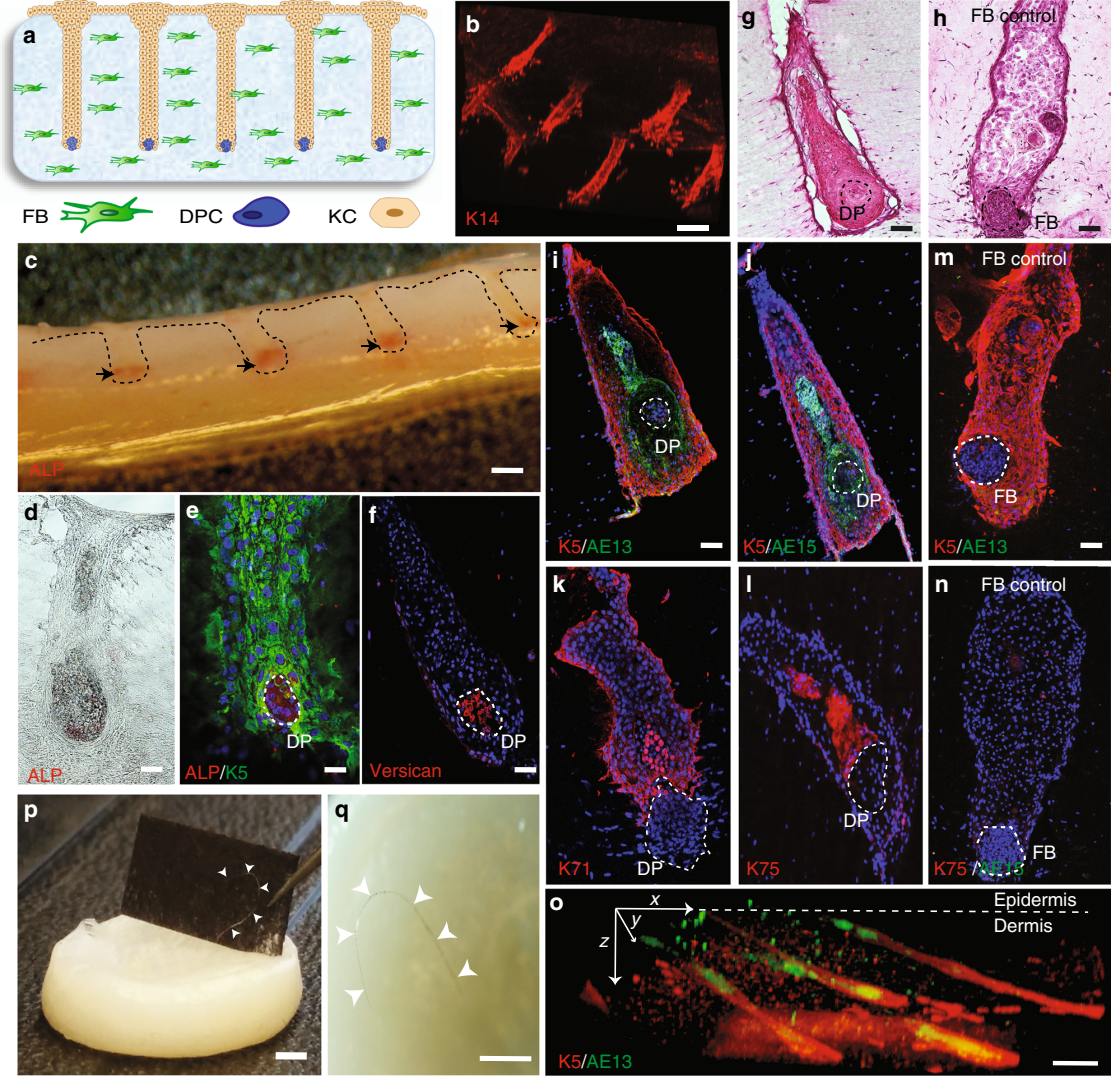
Differentiation of human keratinocytes into specific hair follicle lineages in HSCs. **a** Schematic and **b** 3D-reconstructed image of K14-positive cells showing microwells filled with KCs after a day of culture. **c** Cross section of the whole HSCs with the ALP-active labeled cells at the tip of the microwells (black arrows). Bright-field (**d**) and **e**, **f** immunofluorescent images of HSCs reveal the physiological conformation of cells where ALP-active and VCAN expressing DPCs (dashed circles) are engulfed by K5-positive KCs after a day of culture, forming HFUs. H&E staining of HSCs depicted the morphological changes of KCs above DPC aggregates (**g**), which was not observed in HSCs with FB aggregates (**h**). KCs in the HSCs expressed specific hair lineage and differentiation markers K5 and AE13 (**i**), AE15 (**j**), K71 (**k**), and K75 (**l**), which was not observed in HSCs with FB aggregates (**m**, **n**) after a week of culture. **o** Culture of HSCs for 3 weeks resulted in elongated hair follicles and improved organization of inner and outer root sheaths as shown by the K5 and AE13 markers, respectively. **p**, **q** Prolonged culture period led to hair fiber formation and protrusion from the HSCs (arrow heads indicate the hair fiber). Scale bars for (**b**), (**c**) and (**o**−**q**) are 2 mm and for (**d**−**n**) are 100 µm (*n* = 9; three technical and three biological replicates)

To establish a physiological conformation of cells, we seeded keratinocytes (KCs) over the dermal constructs and allowed the cells to settle down and fill up the microwells, engulfing the DPC aggregates (Fig. 2a, b) and forming an overlying column of KCs that resembled a hair follicle-like unit (HFU). We confirmed the sustained activity of ALP in DPCs after the addition of the KCs. Cross-sections of the whole constructs exhibited ALP activity at the base of the HFUs (Fig. 2c, d), where the DPC aggregates with ALP activity and VCAN expression were surrounded by K5-positive KCs (Fig. 2e, f). Reflecting the physiological proximity and conformation of epidermal and mesenchymal cells, KCs directly above the DPC aggregates showed differentiated morphology resembling KCs in the HF after a few days in culture (Fig. 2g), whereas no morphological changes were observed with the FB control (Fig. 2h). Culturing the 3D skin constructs for a week resulted in the differentiation of KCs into specific hair lineages, including Keratin-5 (K5) (an outer root sheath marker), AE13, AE15 and K71 (inner root sheath markers), and K75 (a hair medulla and companion layer marker) (Fig. 2i−l). When the same experiments were performed with FB aggregates as a control, KCs remained in their undifferentiated state by retaining the expression of K5, and failure to express the hair lineage markers, AE13 or K75 (Fig. 2m, n). In these analyses, we did not observe differentiation of KCs into the sebocyte lineage as evident by the absence of Oil-red-O staining within the follicular structures (Supplementary Figure 2).

Extending the culture period from 1 week to 3 weeks in vitro led to the elongation of the HFs down into the dermis and a better organization of the inner and outer root sheath layers (Fig. 2o). Interestingly, the HFs spontaneously repositioned their orientation from an initial angle of 90 degrees to a more physiological obtuse angle (>120 degrees) after 3 weeks of culture in vitro (Fig. 2o). Remarkably, in some of our constructs, we observed hair fibers protruding from the surface of the HSCs (Fig. 2p, q). Although at this stage, this process was relatively inefficient and was only observed approximately in one out of three constructs. However, to our knowledge, this is the first demonstration of the generation of human HFs in HSCs in an entirely ex vivo context.

### Improvement of HF induction by genetic reprogramming

In our HSCs, we found that the ratio of the HFUs demonstrating HF differentiation to the total number of HFUs (success rate) was as low as 19%, perhaps due in part to incomplete restoration of DPC hair inductive gene signature (~22%) achieved solely through 3D aggregate formation^7^. Therefore, to further enhance hair inductivity, we leveraged two MR genes, *Lef-1* and *Fli-1*, which we previously identified as the key regulators of the intact DPC gene signature^7^. Interestingly, our previous systems biology approach showed that both spheroid culture of DPCs and *Fli-1* overexpression restored very similar expression profiles^7^. In contrast, *Lef-1* targeted genes that were not restored by spheroid culture, suggesting that *Lef-1* overexpression and spheroid culture are complementary for restoration of the DPC hair inductive transcriptional signature.

**Fig. 3:**
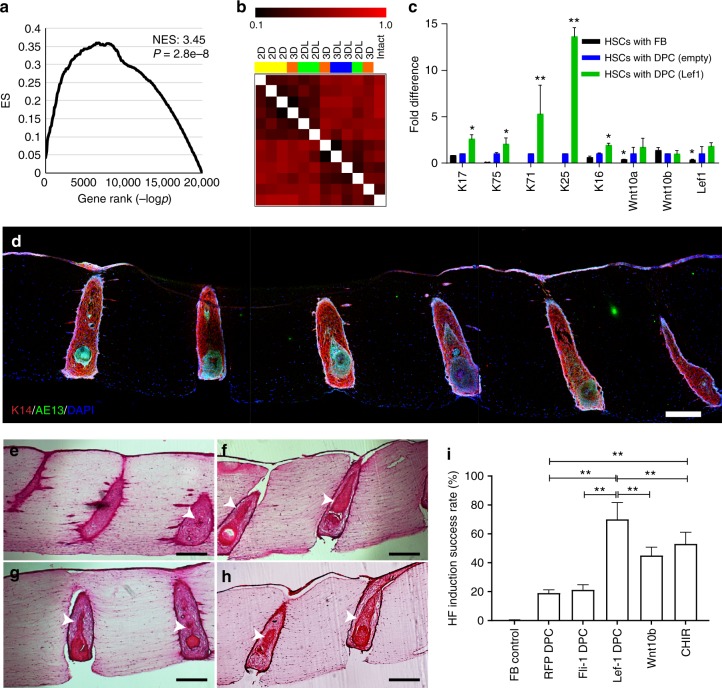
Overexpression of the master regulator gene *Lef-1* in DPCs enhances the efficiency of hair follicle induction in HSCs. **a** GSEA of the RNA-sequencing data of *Lef-1*-transfected DPCs revealed that *Lef-1* overexpression significantly overlaps the downstream network genes previously predicted by ARACNE algorithm (NES, normalized enrichment score). **b** Gene distance matrix showing the individual and synergistic effects of *Lef-1* overexpression and 3D-spheroid culture in DPC transcriptional signature (2D: adherent culture; 3D: spheroid culture; 2DL: adherent culture of *Lef-1*-transfected cells; 3DL: spheroid culture of *Lef-1*-transfected cells). **c** Expression levels of specific hair follicle layers and Wnt-signaling genes determined by qPCR from the total mRNA of HSCs containing *Lef-1*-transfected DPCs (green), empty vector-transfected DPCs (blue) and FBs control (black). Fold difference values are based on the empty vector-transfected DPCs. **d** Low magnification image demonstrating expression of AE13 in HSCs generated using *Lef-1*-transfected DPCs and the high efficiency in HF lineage differentiation. Histological H&E staining of the constructs generated by empty vector-transfected DPCs (**e**), *Lef-1*-transfected DPCs (**f**), Wnt10b-treated DPCs (**g**), and CHIR99021-treated DPCs (**h**) (arrows showing differentiated KC morphology). **i** Success rate, defined as the ratio of the HFUs exhibiting hair follicle differentiation to the total number of HFUs, for HSCs with DPCs at different treatment conditions compared to FB control (*n* = 3 with cells from three donors, *p < 0.05 and **p < 0.005 from two-tailed *t* test); Center values and error bars are defined as means and s.e.m.; Scale bars are 300 µm

To test this hypothesis, we performed transient transfection of *Lef-1* in cultured DPCs (passage 3) (Supplementary Figure 3), and then formed spheroids with *Lef-1*-transfected cells. We compared their gene expression profiles to DPCs transfected with an empty vector control, as well as freshly isolated DP using RNA-sequencing. The *Lef-1* downstream network genes predicted previously by the ARACNE algorithm were experimentally validated by overexpression of *Lef-1* in cultured DPCs via gene set enrichment analysis (GSEA), yielding a normalized enrichment score (NES) of 3.45 and a *p* value (two-tailed *t* test) of 2.8×10^-4^ (Fig. 3a). Gene distance matrix (Fig. 3b) and hierarchical clustering analyses (Supplementary Figure 4) showed that *Lef-1* overexpression and 3D-spheroid culture synergistically restored the intact DPC gene signature. Skin constructs generated with DPCs overexpressing *Lef-1* resulted in significant increase (up to 13-fold) in the expression of the specific hair lineage genes, including the outer and inner root sheath (K17, K71, K25), and hair companion and medulla markers (K75), compared to the DPCs transfected with empty vector (Fig. 3c). HSCs made with FB aggregates did not exhibit any expression of the inner root sheath genes, K71 and K25, as expected. The fold differences in Fig. 3c were normalized to the expression of the genes in HSCs based on the empty vector-transfected DPCs. Similarly, *Lef-1* overexpression in DPCs markedly increased the percentage of the HFUs with KC differentiation (Fig. 3d), increasing the success rate of HF induction markers from 19 to 70% (Fig. 3e, f, i).

To extrinsically recapitulate the role of Lef-1, we treated the constructs with recombinant human Wnt10b and CHIR99021, a small molecule that activates Wnt-signaling through inhibition of GSK3. Treatment with these factors (instead of *Lef-1* overexpression) also increased the success rate up to ~50%, albeit lower than that was achieved with *Lef-1* overexpression, highlighting the Wnt-signaling-independent role of cellular reprogramming (Fig. 3g−i).

The exogenous expression of the other MR gene, *Fli-1*, resulted in significant reprogramming of the gene expression in cultured DPCs (Supplementary Figure 5a) and upregulated several *Fli-1* downstream network genes, such as collagen 5 (*COL5*) and collagen 6 (*COL6*) (Supplementary Figure 5b), in agreement with our previous gene network analyses. Although *Fli-1* overexpression in DPCs induced HF differentiation in HSCs (Supplementary Figure 5c) with a success rate of 21%, it did not significantly improve the induction achieved by empty-vector-transfected DPCs (Fig. 3i). Overall, these data supported our previous predictions that *Fli-1* overexpression and spheroid formation have an overlapping effect in DPC reprogramming, whereas *Lef-1* overexpression complements aggregate formation and significantly improves the restoration of DPC hair inductivity.

### Generation of vascularized HSCs with high-follicle density

**Fig. 4:**
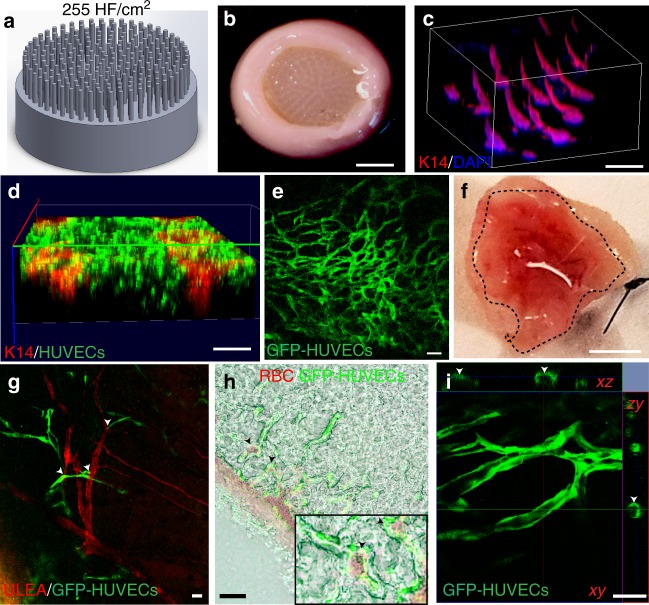
Vascularization of high hair-follicle-density HSCs for efficient engraftment. **a**, **b** HSCs were generated using the molds that have 255 HF per cm^2^ to promote hair growth in grafts. **c** 3D-reconstructed wholemount image of the HSCs showing 3D conformation of K14-positive cells at the high hair follicle density. GFP-tagged HUVECs that were encapsulated in the dermal compartment with the fibroblasts closely surrounded the K14-positive cells in the hair follicle structures (**d**) and formed capillary-like networks after 3 days of culture (**e**). **f** Bottom view of the explanted prevascularized HSCs (dashed circle) with GFP-HUVECs showing promoted host vascularization and blood supply to the grafts (*n* = 9; three technical and three biological replicates); **g** HSCs grafted onto mice were revascularized by the host vessels where GFP-tagged HUVECs (green) formed capillaries that are well-organized around the mouse vessels (*GS*-*IB*_*4*_ staining in red; arrow heads show the proximity of mouse and human vessels) and red blood cells (RBCs) (**h**), (arrow heads show host RBCs near human blood vessels and lower panel shows a magnified area of the image); and exhibited lumen formation (arrow heads show the lumen) (**i**). Scale bars for (**b** and **f**), (**c**, **d**), and (**e**, **g**, **h** and **i**) are 4 mm, 2 mm, and 100 µm, respectively

We next examined the capability of our HSCs containing *Lef-1*-transfected DPCs to grow human hair in vivo by grafting them onto immunodeficient nude mice. To more closely recapitulate the physiological hair density in human scalp^13^, we increased the HF density from 81 HF to 255 HF per cm^2^ (Fig. 4a−c and Supplementary Movie 1) using 3D-printed high HF density molds. The first set of experiments did not result in hair formation; instead, we observed substantial necrosis at the center of the grafts due to lack of host vascularization in the grafts (Supplementary Figure 6a, b), consistent with our previous work^5^.

To generate a vascular bed, we encapsulated GFP-tagged human umbilical vein endothelial cells (HUVECs) in the dermis of our HSCs together with the dermal fibroblasts (Fig. 4d). Culturing the constructs at a HUVEC to FB ratio of 16:1, we induced spontaneous capillary formation in the dermis (Fig. 4e). Immunofluorescent wholemount imaging of the constructs revealed that these capillary-like structures were in close proximity to the HFs (Fig. 4d). Grafting the vascularized HSCs onto mice promoted host vascularization into the grafts (Fig. 4f). GFP-tagged HUVECs formed more organized and elongated networks upon grafting, which were located in close proximity to the host red blood cells and newly formed host vessels of the mice that were labeled with mouse-specific rhodamine-conjugated *Isolectin Griffonia simplicifolia* (*GS*-*IB*_*4*_*)* (Fig. 4g, h), and also exhibited lumen formation (Fig. 4i).

### Hair induction in HSCs grafted onto mice

**Fig. 5:**
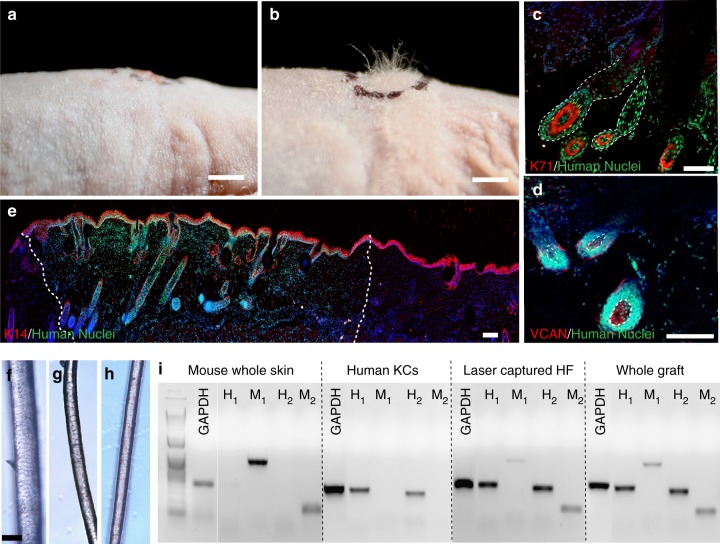
Induction of human hair growth in immune-deficient nude mice. **a**, **b** Engraftment of high follicle-density HSCs onto immune-deficient nude mice led to hair growth in the grafts after 4–6 weeks (**b**), which was not observed in the HSCs prepared with FB aggregates as a control (**a**) (*n* = 10; five biological and two technical replicates per each condition). **c**, **d** Human-specific nuclear staining (green) indicated that the de novo hair follicles marked with K71 (**c**) (HF boundaries circled in dashed line) and DPCs marked with VCAN (**d**) are comprised of human cells (DPs circled in dashed line). **e** Low magnification image of the explanted grafts revealed the boundaries (dashed line) between the mouse and human tissues as marked by the human nuclear staining. Bright-field microscopy of unpigmented terminal human hair (**f**), engineered human hair in the grafts (**g**), and unpigmented human vellus hair (**h**) showed morphological similarities between human hair and engineered hair. **i** PCR was performed with two sets of primers specific to human and mouse cells using the RNA of the laser captured hair follicles from the grafts in comparison to whole grafts, mouse skin, and cultured human KCs. Scale bars for (**a**, **b**), (**c**−**e**) and (**f**−**h**) are 2 mm, 200 µm, and 50 µm respectively

Four to five weeks after grafting our vascularized HSCs at a high follicle density of 255 HF per cm^2^ onto immunodeficient nude mice, we observed substantial hair growth in the grafts, whereas the HSCs prepared with FB aggregates did not induce hair formation (Fig. 5a, b). In the grafting experiments, we used ten mice per condition. Our vascularization strategy enabled the survival of seven out of ten grafts, both for HSCs prepared with DPCs as well as FBs as a negative control. Grafts from four out of these seven mice successfully generated human HFs, whereas none of the seven mice in the FB control experiment induced hair formation. Immunostaining with a human-specific nuclear antibody confirmed that these HFs were comprised of differentiated human KCs and DPCs, as indicated by K71 (Fig. 5c) and VCAN (Fig. 5d) staining, respectively. In addition, we did not detect any presence of dermal sheath (DS) around the engineered HF, as evidenced by negative SMAα staining (Supplementary Figure 7a), whereas the mouse hair showed expression of SMAα around DPCs marked with Lef-1 (Supplementary Figure 7b). These data suggest that DPCs and KCs are sufficient to grow de novo HFs without the presence of DS, although DS may be required for the long-term support and maintenance of the HFs at later stages. Low magnification images of the human-specific nuclear staining and K14 (stain appears in multiple layers due to tilted angle of the constructs) clearly delineated the edges between the host and grafted skin, and most of the regions of the mouse skin lacked HFs (Fig. 5e). Our grafts have only a slightly thicker epidermis than the mouse, consistent with our grafting strategy and timing, in which we chose to not cornify the epidermis at high calcium conditions to avoid any interference with HF differentiation (as opposed to epidermal differentiation) prior to grafting. Therefore, further epidermalization and cornification seen in Fig. 5e took place after grafting onto mouse, suggesting that epidermal differentiation could be controlled by the host microenvironment.

Microscopic bright-field images of the hair fibers showed that engineered hair (Fig. 5g) closely resembles the morphology of human terminal hair (Fig. 5f); and moreover, that it represented an intermediate thickness between human terminal and vellus hair (Fig. 5h). To confirm the human origin of the de novo HFs, we selectively isolated the RNA of the cells from the HFs within the grafts using laser capture microdissection (Supplementary Figure 8a, b) and performed PCR using human vs. mouse-specific primers (see Supplementary Table 1)^15^. The PCR analyses with both sets of primers confirmed that HFs in the grafts highly expressed the human-specific sequence, whereas the control whole mouse skin tissue only expressed mouse-specific genes (Fig. 5i), demonstrating that the HFs induced in the grafted HSCs are indeed derived from human cells. We also observed low levels of mouse-specific gene expression in the HFs, consistent with presence of some cells in the grafts that are negative for human-specific nuclear staining (Fig. 5c−e). These cells may represent recruitment of mouse vasculature and infiltrating cells to the microenvironment of the HFs, since the levels of mouse-specific gene expression were much higher in the whole grafts compared to the laser captured HFs (Fig. 5i).

## Discussion

Tissue engineering of human HFs has been a long-standing challenge and its progress has lagged behind other lab-grown tissues, such as vasculature^16^ and intestinal epithelium^17^. This is mainly due to the lack of availability of a platform that can successfully recapitulate the microenvironmental cues required to maintain the requisite cell interactions for hair neogenesis. Recently developed 3D-printing technology has allowed for creation of structures with high aspect ratios (length to width ratio: ~100 for human HFs^13^), which was not possible with previous microfabrication techniques, such as soft lithography. Utilizing this capability, we generated HF-like microwells in 3D-reconstructed dermis where genetically/extrinsically reprogrammed cells could be arranged easily into a physiologically relevant conformation, forming HFUs. This biomimetic approach led to KCs differentiation into specific hair lineages and allowed us to generate human HFs within HSCs in an entirely ex vivo context.

Since the animal body offers a physiological environment for the development and engraftment of animal/human tissues, the first demonstration of fully regenerated organs typically involves transplantation of tissues onto animals. For instance, creating de novo adult HFs from cultured cells dates back to 1984, where cultured rodent HF DPCs induced hair growth in recipient skin^18^. The same group was the first to achieve hair growth in reconstructed follicles in culture^19^. Similarly, two recent studies established the growth of mouse hair in vivo through encapsulation of mouse iPSCs^20^ or mouse adult DPCs and epidermal cells^21^ in hydrogels followed by transplantation of these in vitro-conditioned structures into mice. Another recent study also demonstrated the spontaneous formation of HFs in embryoid bodies of mouse iPSCs^22^.

Despite these successes, significant interspecies differences exist between human and mouse HFs not only in their hair cycles, stem cell characteristics, and hormonal dependencies^23^, but also in the hair inductive properties of their dermal cells. One striking difference is that cultured rodent DPCs can self-aggregate when transplanted onto rodent skin^24^; however, this aggregative behavior is not observed in human DPCs^25^. In a study from Toyoshima et al., human HFs were induced in mice by using a hair germ-like 3D culture method by mixing bulge region-derived epithelial cells and scalp HF-derived intact DPCs^6^. This study represented a valuable proof-of-concept that human DPCs can generate HFs in vivo. However, to translate and scale such an approach into a clinical reality, these cells must be expanded in vitro for several passages, which causes a rapid loss of hair inductive gene signature in DPCs^7,8^.

We recently addressed this issue by 3D-spheroid culture of cells and thereby restored 22% of the hair inductive DPC gene signature. Subsequently, other groups also reported the use of this method to induce HFs in mice, albeit inefficiently^11^. To enhance the efficiency of hair induction properties, in this study, we combined genetic and microenvironmental reprogramming strategies by overexpressing the MR gene *Lef-1* in combination with spontaneous DPC spheroid formation in the HSCs, which resulted in 70% success rate of HF formation ex vivo, compared to only 19% with the empty vector-transfected DPCs.

Vascularizing and grafting these HSCs led to efficient induction of human HF formation in mice. Although our MR strategy significantly enhanced the hair lineage differentiation in vitro, the efficiency of hair growth with a fully developed hair shaft was much higher in the grafted HSCs compared to in vitro cultured HSCs, reflecting the limitations of the in vitro/ex vivo approach in terms of the culture conditions. This will be addressed in the future by leveraging appropriate growth factors, small molecules or proliferation enhancers.

Tissue-engineered human HFs have many compelling applications including: (i) skin replacement therapy for full-thickness skin loss; (ii) hair restoration surgery; (iii) substituting/complementing animal models of hair disease for drug development; and (iv) testing of cosmetic products. Our method has several advantages over previous approaches, which rely on spontaneous formation of HFs^6,22^, especially in terms of the ability to control the pattern and density of the follicles, and to use expanded primary cells (>passage 3) yielding sufficient number of cells for any type of application. For example, for third degree burn patients that require full-thickness skin grafts, spatial control over HFs may facilitate recreating the variations in hair density at the site of transplantation (e.g. 292 HF per cm^2^ in forehead vs.18 HF per cm^2^ in forearms^13^). Interestingly, Plikus et al. recently showed that BMP signaling from HFs can convert myofibroblasts into adipocytes and potentially reduce scar formation after wounding^26^. The presence of HFs in our HSCs may enable this function by promoting adipocyte differentiation in the wound bed, although this requires further investigation. In addition, the formation of a scar or nonfunctional skin is typically observed in skin grafts due to poor viability and integration of HSCs^27,28^. Our ability to vascularize hair-bearing HSCs will significantly promote the viability of both the skin and hair tissues, constituting a groundbreaking innovation for regenerative skin therapies.

Robotic hair restoration surgery has revolutionized modern hair transplantation by allowing for performing repetitive maneuvers within high precision and speed^29^. Hair restoration surgery to treat male and female pattern baldness usually requires 100 cm^2^ of donor tissue site to deliver nearly 2000 grafts (~4400 hairs) per patient. Using our strategy, we typically generate 15 million DPCs (passage 3) from a strip of donor tissue as small as 0.5 cm^2^, yielding enough cells to produce more than 5000 HFs in our HSCs, making it a feasible and efficient strategy to integrate with hair restoration therapy. The improvements of these robotic systems to harvest hair follicular units (groups of 1–4 hairs), as opposed to single hairs, yield significantly more hair growth per harvest^30^. Using 3D-printing approaches, our goal is to engineer HFs as follicular units and/or in desired patterns that can be integrated with surgical robots and facilitate effective hair transplantation surgery. Moreover, from a technical perspective, harvesting HFs with an obtuse angle, compared to 90 degrees, is more challenging and typically requires larger incisions^31^. In our 3D-printing approach, we used a starting HF angle of 90 degrees, which is then self-reorganized within the constructs above 120 degrees after 3 weeks of culture or skin grafting. However, for hair restoration applications, we envision that HFUs in our hair constructs can be harvested as soon as a week after their preparation, providing a more practical starting material to harvest HFs with a straight incision perpendicular to the epidermis.

Organ culture of HFs is still the current gold standard for drug testing on human HFs. This method has several limitations including its low-throughput, dependence on fresh, living HFs from human donors, difficult to standardize due to wide inter-individual variation in rates of hair growth (due to different human donors), inability to capture the signaling from the dermis (e.g. dermal fibroblasts and endothelial cells), and the short life-span of the cultures. Our ability to incorporate engineered HFs into HSCs represents the first major step to circumvent some of these limitations. We are currently able to make nine skin constructs containing 255 HFs in a six-well-plate format starting from only one HF donor tissue, which is a substantial improvement on the throughput aspect of drug testing (1:1 in the organotypic assay). In addition, the contribution of the dermal compartment on the HFs can potentially be examined especially with the addition of other dermal components in the future such as immune cells.

One of the immediate extensions of our approach will be the incorporation of melanocytes to generate pigmented HFs. We previously derived melanocytes from iPSCs, and showed their capability to transfer their melanin to neighboring keratinocytes^32^. Moreover, in our analyses we did not examine the capability of these engineered HFs to undergo the hair cycle, which relies on the presence and maintenance of a reservoir of stem cells in our HSCs. Recently, De Luca’s group demonstrated that holoclonal epidermal stem cells^33^, a subpopulation of cultured KCs with a high self-renewal capacity, are capable of regenerating the entire human epidermis^34^. The ability to maintain the holoclonal epidermal stem cells in a feeder-free environment over several passages, as well as within HSCs over long periods, could provide greater regenerative capacity to engineered HFs for translational purposes. Alternatively, incorporation of HF stem cells^35,36^ or iPSC-derived folliculogenic epidermal cells^37^ separately into HSCs could potentially establish the hair cycle. However, adding a stem cell niche, such as the bulge region, containing HF stem cells into our constructs will require better resolution in the spatial control of cells, which is a current technical limitation of our method utilizing 3D-printed molds. In the future, 3D-bioprinting technology operating at a single cell resolution may permit the inclusion of other cell types, such as stem cells and melanocytes, to generate cycling and pigmented HFs.

A recent study revealed that hair organoid formation of mouse cells in vitro follows a different self-organization mechanism than what occurs during hair morphogenesis, despite resulting in successful hair growth in vivo^21^. In agreement with this finding, our approach exploits the epidermal−mesenchymal interactions during hair development while synthetically guiding the physiological conformation and reconstituting the gene signature of cultured cells to induce human hair growth in vitro and in mice.

Our novel biomimetic developmental tissue engineering strategy represents a crucial step forward in generating a truly functional human skin and marks a dramatic conceptual advance in regenerative medicine approaches to disorders of the skin and HF, as well as improving the outcome of severe skin injuries leading to disfiguring scars. Adaptation of this new technology by hair researchers, hair restoration surgeons and the pharmaceutical and cosmetic industries will have overwhelming implications in the maintenance and regeneration of this complex human tissue.

## Methods

### Cell isolation and culture

Neonatal dermal keratinocytes and fibroblasts were isolated from human foreskin and cultured in CnT-07 (CELLnTEC) up to passage 3 (P.3) and in Dulbecco Modified Eagle Medium (DMEM) with 10% Fetal Bovine Serum (FBS) up to P.5, respectively. GFP-tagged HUVECs (Angio-proteomie) were cultured in tissue culture flasks coated with a quick coating solution (Angio-proteomie) and maintained in endothelial cell growth medium (EGM, Angio-proteomie). DPCs were isolated from discarded scalp tissues from hair restoration surgery (kindly provided by Dr. Robert Bernstein) using microdissection^38^, and cultured in DMEM with 10% FBS up to P.3. Culture medium was changed every other day for all cell types. The cells were kept in a humidified incubator at 37 °C and 5% CO_2_.

### Generation of HSCs with hair follicle patterns

All 3D-printed molds were designed and drawn using the computer-aided design (CAD) software, Solidworks. Each HF-like extension on the molds was 500 μm in diameter and 4 mm in length. The molds with varying hair densities (19, 81, 255 HFs per cm^2^) were 3D-printed using Objet24 3D-Printer (Stratasys) which uses a UV-curing material VeroWhite (Stratasys). 3D skin constructs were generated in six-well-plate transwell inserts similar to the method described previously^39^. The dermal compartment was prepared by adding 4 mL of type I collagen matrix containing 1.25 × 10^5^ fibroblasts per ml into the transwell inserts and polymerized around the 3D-printed HF molds placed on top of the gel at 37 °C for 30 mins. After complete polymerization, the molds were removed and 100 μl of DPC cell suspension at a density to give 3000 DPCs per microwell was added on top of the gel (e.g. 7 million cells per ml for 255 HF per cm^2^). The constructs were cultured overnight in DMEM with 10% FBS for aggregate formation, after which 1 million KCs were added on top of the gel. The constructs were maintained in low calcium epidermilization medium^39^ submerged for 1–3 weeks.

### Vascularization of the HSCs

GFP-tagged HUVECs were encapsulated in collagen type I gel at a concentration of 2 million cells per ml to give a HUVEC to FB ratio of 16:1. After removal of the 3D-printed molds, HSCs were first left in EGM-2 medium kit supplemented with growth factors (Lonza) for 3 days for capillary formation. The DPC and KCs cell seeding were performed as described above. After a day of culture in a 1:1 mixture of EGM-2 and the epidermilization medium, the constructs were either imaged or used for grafting experiments.

### Histology, immunostaining, and imaging

For immunostaining and histological staining, samples were cut in half and embedded in paraffin wax or cryopreserved in OCT solution. Formalin (10%)-fixed, paraffin wax-embedded tissues were cut (20 µm) onto poly-l-lysine-coated slides, dried overnight at 55 °C, dewaxed in xylene and rehydrated through a graduated ethanol series (100, 95, 70%) and distilled water (dH_2_O). Samples were rinsed briefly with phosphate-buffered saline (PBS) and blocked using 2% fish skin gelatin (Sigma) in PBS containing 0.025% Triton-X-100 for 90 min at RT. Samples were incubated with primary antibodies (see the list of antibodies and dilutions used in Supplementary Table 2) overnight at 4 °C. After washing with PBS, samples were incubated with fluorophore-conjugated secondary antibodies (Donkey anti-Rabbit 594, and Goat anti-mouse 488, 1:700, Invitrogen) for 2 h at RT. Slides were covered with cover-slips using mounting medium containing 4′,6-diamidino-2-phenylindole (DAPI) (Vectashield) and examined using a Zeiss LSM 5 Exciter confocal laser scanning microscope. ALP staining was performed using the VactaShield ALP activity kit following the manufacturer’s instructions. Wholemount tissue staining was performed similarly to the staining of the sections except that the tissues were incubated in the primary antibody for 3 days and the secondary antibody overnight. The tissue was cleared by benzyl alcohol to benzyl benzoate mixture (1:2) before imaging.

### Haematoxylin and eosin staining (H&E)

Dewaxed 20 µm sections were stained with Mayer’s Haematoxylin (Sigma) at RT for 3 min. Blue staining was performed by rinsing in tap water, while differentiation was achieved by rinsing in 1% acid ethanol. Counterstaining was performed by rinsing with eosin (Sigma) for 30 s and dehydration was performed by sequential washing with 95% ethanol, 100% ethanol and Histo-Clear (National Diagnostics). Slides were covered with cover-slips with DPX (Agar Scientific) and examined by light microscopy using a Zeiss Axioplan 2 microscope.

### HSC engraftment onto mice

All experimental animal protocols were approved by the Institutional Animal Care and Use Committee at Columbia University Medical Center. 0.8 cm^2^ of skin was removed from the dorsal antero-posterior midline surface of 8–10-week-old, male immunodeficient nude mice (athymic nude, Crl:NU(NCr)-Foxn1nu, Charles River, Wilmington, MA) using the pinch cutting technique. A silicone chamber with a diameter and height of 1 cm was inserted underneath the skin on the back of the mouse. Only one chamber was placed on each mouse. The chamber was hat shaped, with a hole in the top of the hat. The HSCs were placed into the chambers and maintained for 5 days by adding epidermalization medium everyday into the chamber. Subsequently the chamber was removed and the HSCs were secured with four sutures (7–0 Nylon) in a simple interrupted pattern around the edge of the graft. A Bandaid was then wrapped around the mice (an OpSite Flexifix Transparent Film) to hold the graft in place. The mice were euthanized after 4–6 weeks for analyses. To visualize the vascularization, the mice were injected intravenously through tail vein with mouse-specific rhodamine-conjugated *GS*-*IB*_*4*_ (1:10; Invitrogen) solution 20 min prior to euthanasia^40^. The engraftment experiments were performed using five biological replicates (separate batches of HSCs) on two mice per each biological replicate (*n* = 10) per condition.

### Transfection of DPCs

The plasmids pBABE-puro LEF1 (Addgene, #27023) and pCMV3-FLI1 (Sino Biological, HG14507-UT) were purchased. DPCs at passage 3 were seeded in six-well plates at 100,000 cells per well and cultured overnight prior to transfection. Lipofectamine P3000 transfection reagent was used to transfect the cells. In each well, the cells were maintained in 250 µl of P3000 with 2.5 µg of DNA overnight and the medium was changed to normal culture medium for further use or analyses.

### RNA-sequencing

Total RNA was isolated from cells maintained at indicated conditions by using the RNeasy Mini Kit (Qiagen) in accordance with the manufacturer’s instructions. Only the total RNA that received an RNA integrity number (RIN) of 9 or higher was submitted for RNA-sequencing to the Columbia University sequencing facility. Samples were processed at 30 million reads per sample. For the sequencing analysis, reads were aligned to the human reference genome (hg19). Gene counts were calculated using HTSeq and were used as an input for differential gene expression analysis with DESeq (version 1.20.0). Comparing the 2D cultured DPCs with the intact DPCs, genes with a *p* value (two-tailed *t* test) of less than 0.05 as well as a twofold change were chosen for further analysis. Gene expression data of the samples clustered in heatmaps and gene distance matrix were performed using unsupervised hierarchical gene clustering analyses.

### Gene set enrichment analyses

We previously identified MRs of DPC gene signature by interrogating the skin transcriptome for panel enrichment and associations of differential expression using both Fisher’s Exact Test and GSEA, to identify transcription factors with an enriched set of targets within a particular territory. Here, the GSEA was used to compare the *Lef-1*-targeted genes previously detected by ARACNE algorithm^7^ to the gene expression data found experimentally by RNA-sequencing in *Lef-1*-transfected DPCs. A null distribution for calculating the NES and its associated *p* value was found by label shuffling to randomize the gene rankings. These randomized sets were then used to calculate null ESs over 10,000 iterations to generate a null distribution. The observed leading-edge ES was normalized to this null distribution, and a two-tailed *p* value was generated for this NES.

### Real-time PCR and statistical analyses

To collect RNA from HSCs, the whole tissue was first homogenized by a sonicator in Trizol prior to RNA extraction. cDNA was generated from RNA samples following Invitrogen protocols and using SuperScript III with a mixture of random hexamers and oligo dT primers. qPCR was performed using Sybr Green PCR mix on an Applied Biosystems 7300 Real-Time PCR System. Fold changes were calculated using the delta-delta CT algorithm. Error bars were calculated based on SD across three technical replicates within three biological replicates. An unpaired two-tailed *t* test was used to calculate whether differences between samples, across three technical replicates, were significant, or *p* < 0.05. Primer sequences are available in Supplementary Table 1.

### Laser capture microdissection

Laser capture microdissection was performed at Columbia University Cancer Center Core Facility using a laser capture microscope (Arcturus Engineering, Mountain View, CA, USA). OCT-embedded samples were sectioned on membrane slides at a thickness of 5 μm, fixed in 75% ethanol, and dehydrated in xylene prior to microdissection^41^. The captured tissue was immediately lysed and prepared for further PCR analyses.

### Statistics

The statistical analysis was performed for all the experiments using two-tailed paired *t* test with 95% confidence interval with the software GraphPad Prism. Since we used four different cell types in our engineered tissues and anticipated variabilities between different donors, we chose an adequate sample size by using at least three biological replicates and three technical replicates throughput the study. In particular, the experiments done for establishing HF differentiation in hair constructs in vitro were performed in triplicates using cells from the foreskin of three different donors (biological replicates). qPCR analyses and quantification of the success rate of hair differentiation in vitro was done in triplicates and with three biological replicates for each condition. For the calculations of in vivo success ratio, the engraftment experiments were performed using five biological replicates (separate batches of HSCs) on two mice per each biological replicate (*n* = 10) per condition, yielding a total of 20 mice. The hair regeneration success ratio was based on the vascularized grafts that were viable at the time of harvesting. The researcher who performed the quantification of success rates shown in Fig. 3 was blinded. RNA-seq data collection was performed by a separate core facility at Columbia Genome Center and analyzed without a prior knowledge of the conditions of the samples. No randomization was used. For all statistical analyses, *p* < 0.05 was considered significantly different, where the *p < 0.05 and **p < 0.005. Data were shown as means ± s.e.m.

## Electronic supplementary material

Supplementary Information

Description of Additional Supplementary Files

Supplementary Movie 1

## Data Availability

Data supporting the findings of this study are within this manuscript or available from the corresponding authors upon reasonable request. The RNA-seq data have been submitted to the GEO database under the accession code GSE121112.
