# Supplementary material for: Tissue engineering of human hair follicles using a biomimetic developmental approach
Source: Nat Commun. 2018 Dec 13;9:5301. doi: 10.1038/s41467-018-07579-y (PMC6294003; doi:10.1038/s41467-018-07579-y)
Supplement: Supplementary file 1 — Supplementary Information [file 41467_2018_7579_MOESM1_ESM.pdf]

**Supplementary Information**

# Tissue engineering of human hair follicles using a biomimetic developmental approach

Abaci et. al.

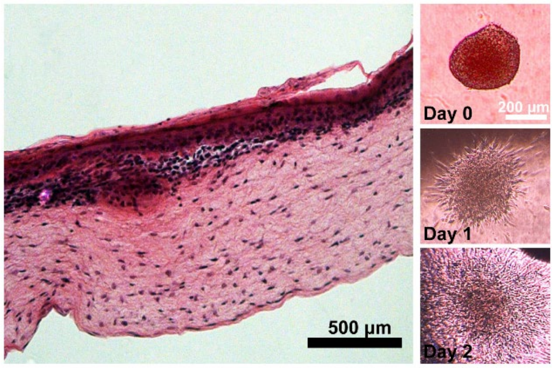


**Supplementary Figure 1. Regression of DPC spheroids in HSCs.** DPC spheroids were produced by the hanging drop method and embedded in HSCs where they gradually regressed and dispersed into the dermal compartment in several days. Scale bars: 500 µm (left panel) 200 µm (right panel)

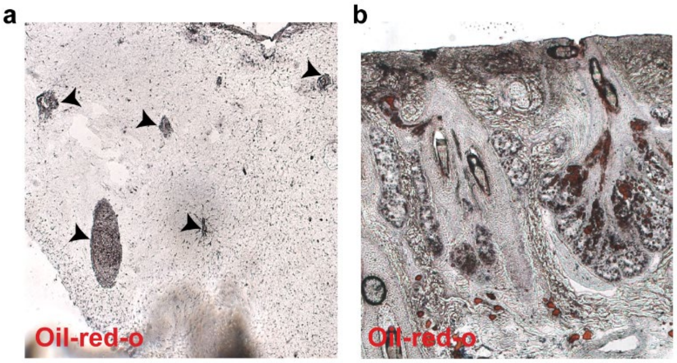


**Supplementary Figure 2. Sebocyte differentiation is not induced in HSCs.** Histological sections of HSCs (arrowheads indicate HF) **(a)** and human scalp tissue **(b)** stained with Oil-red-O.

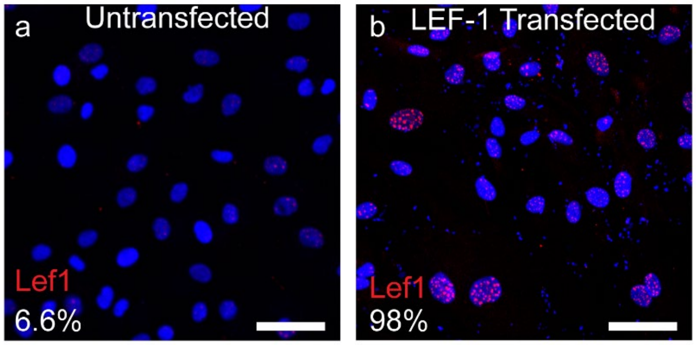


**Supplementary Figure 3. Transfection efficiency of Lef-1.** Untransfected **(a)** and Lef-1 transfected **(b)** DPCs in 2D culture stained with Lef-1 antibody (red) and DAPI (blue). Scale bars: 10 µm

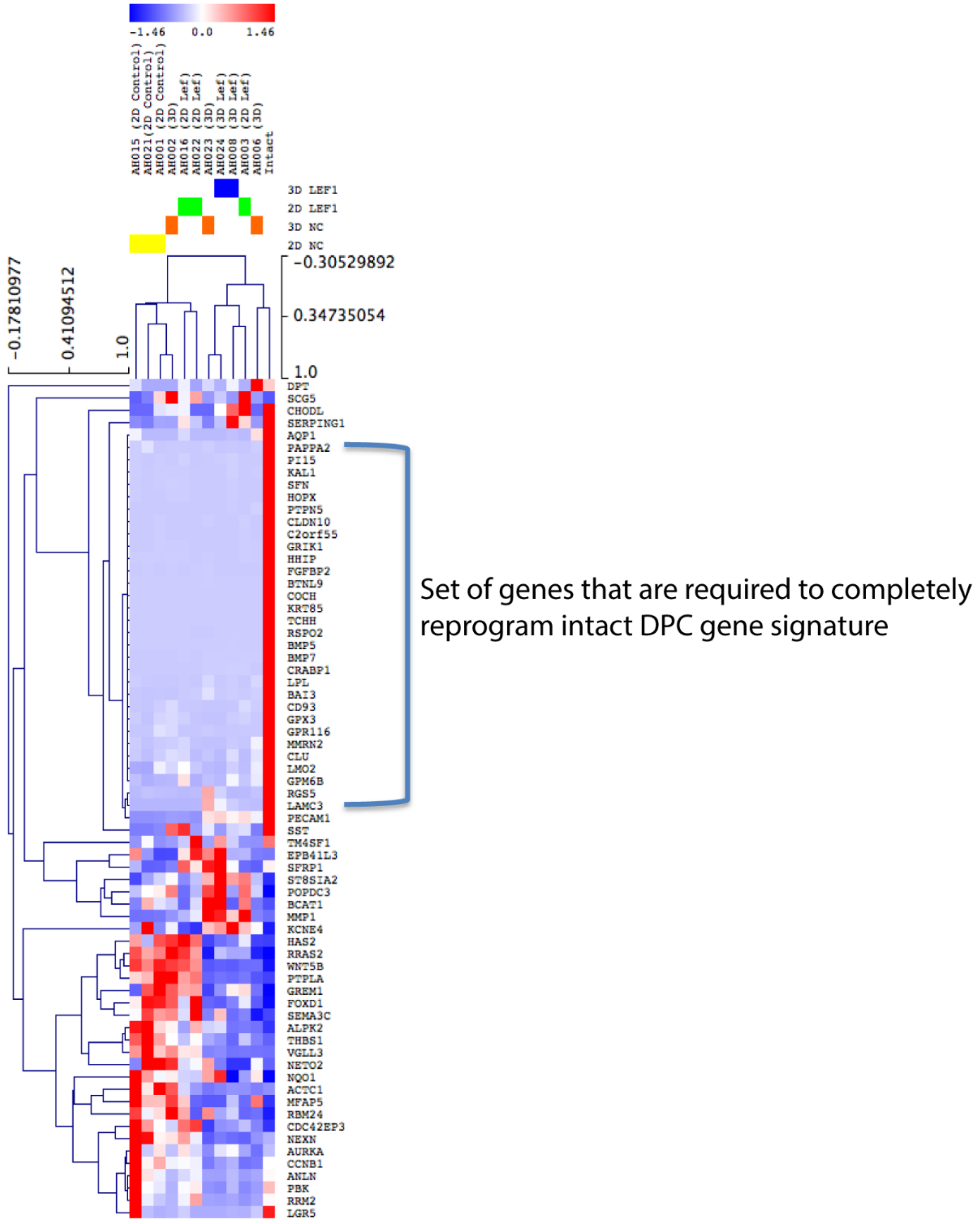


**Supplementary Figure 4. Heatmap and hierarchical clustering analyses of RNA-sequencing data of DPCs cultured in different conditions.** 3D spheroid culture and Lef-1 overexpression both significantly restored intact DPC gene signature.

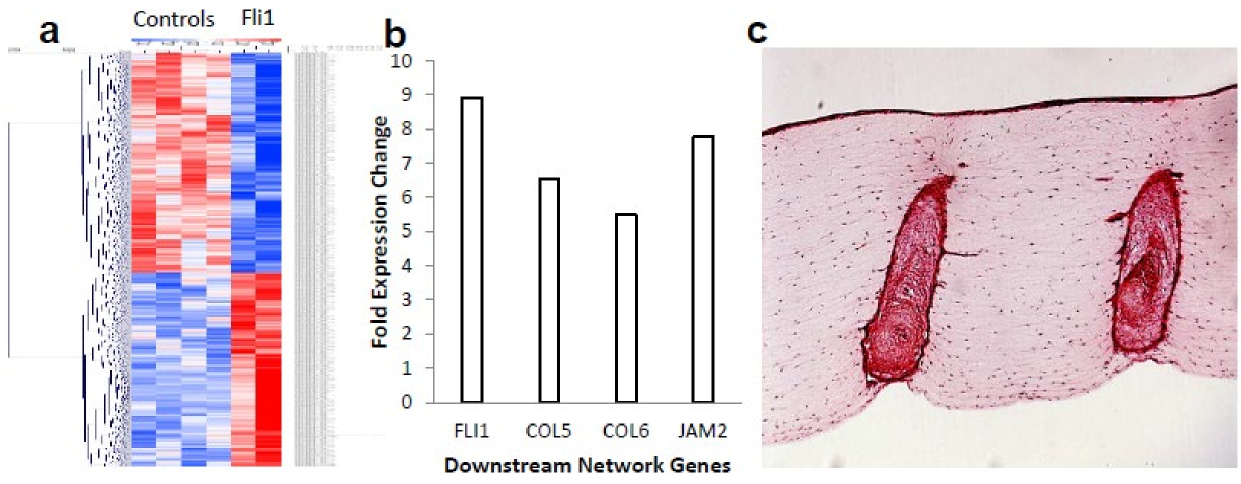


**Supplementary Figure 5. Reprogramming of DPCs by Fli-1 overexpression. (a)** Heat map of DPCs illustrating gene expression profiles of cultured DPCs (passage 3) from three different donors, empty-vector transfected control, and Fli1 overexpressed DPCs from 2 donors. **(b)** RT-PCR of Fli-1 overexpressed cells showed upregulation of Fli-1 downstream network genes as predicted previously by ARACNE algorithm. **(c)** H&E staining of HSCs containing DPCs overexpressing Fli-1.

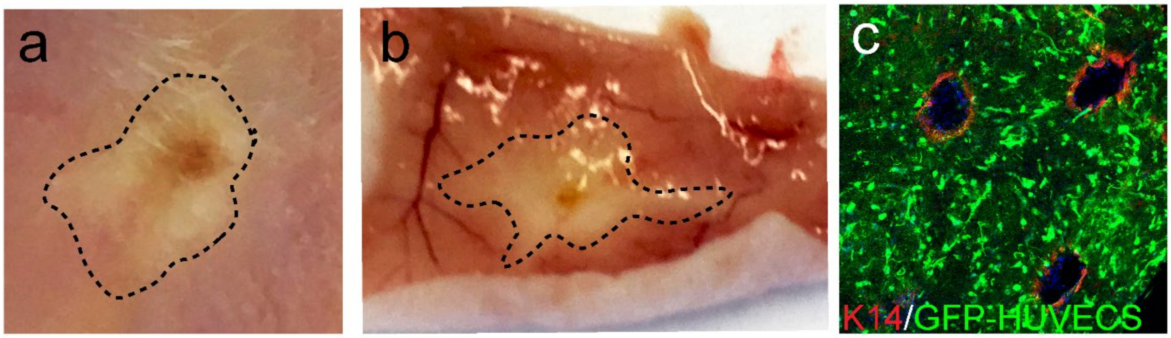


**Supplementary Figure 6. Necrosis in unvascularized hair-follicle-bearing grafts. (a)** Top view of the grafted HSCs showing necrotic tissue at the center of the grafts. **(b)** Bottom view of the explanted HSCs (dashed circle) after 4 weeks showing the absence of blood vessels. **(c)** Encapsulated GFP-HUVECs are in close proximity to the K14-labeled hair follicles in HSCs.

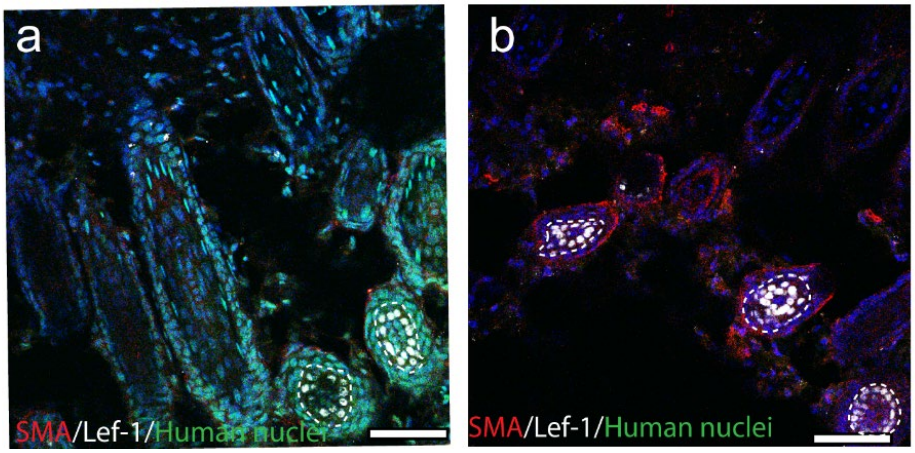


**Supplementary Figure 7. Dermal sheath cells are not present in engineered HFs.** SMAα expression is absent in the engineered human HFs **(a)**, whereas mouse hair follicles express SMAα around the Lef-1-positive DPCs **(b)** (dashed circle indicates the DP compartment). Scale bars are 100 μm

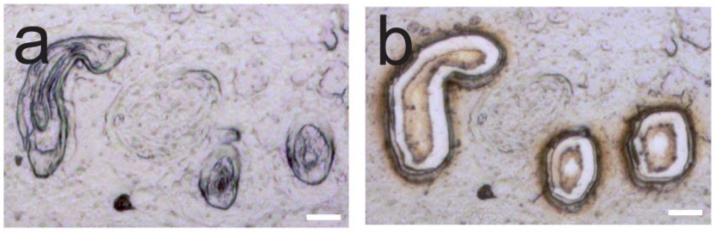


**Supplementary Figure 8. Laser-capture of engineered hair follicles.** Hair follicles in histological sections **(a)** were microdissected using laser capture **(b)**.

**Supplementary Table 1.**

| Primer | Forward Sequence | Reverse Sequence |
|---|---|---|
| K16 | GACCGGCGGAGATGTGAAC | CTGCTCGTACTGGTCACGC |
| K17 | GGTGGGTGGTGAGATCAATGT | CGGCATCCTTGCGGTTCTT |
| K25 | ATGTCTCTTCGACTTTCCAGTGC | GCCACTTCCAATCCCTGAAAT |
| K75 | CCCAGGTCGGTGACACATC | GCAATGTCCTCGTATTGTGCTTT |
| K71 | GCTGCTTACGCCAATAAGGTG | ATCTGAGTGATCTCGGCTTCA |
| Wnt10a | GGAGACTCGCAACAAGATCCC | CGATGGCGTAGGCAAAAGC |
| Wnt10b | CATCCAGGCACGAATGCGA | CGGTTGTGGGTATCAATGAAGA |
| Lef-1 | AGAACACCCCGATGACGGA | GGCATCATTATGTACCCGGAAT |
| Human specific primer 1 | TATTGCAGCCCTAGCAGCACTCCA | AGAATGAGGAGGTCTGCGGC |
| Mouse specific primer 1 | GCACTGAAAATGCTTAGATGGATAATTG | CCTCTCATAAACGGATGTCTAG |
| Human specific primer 2 | TAGACATCGTACTACACGACACG | TCCAGGTTTATGGAGGGTTC |
| Mouse specific primer 2 | ATTACAGCCGTACGCTCCTAT | CCCAAAGAATCAGAACAGATGC |

**Supplementary Table 2**

| Antibody | Company | Dilution | Catalog Number |
|---|---|---|---|
| Keratin 14 | Covance | 1:800 | 905301 |
| Keratin 5 | Covance | 1:800 | PRB-160P |
| Keratin 71 | Covance | 1:100 | NBP2-14176 |
| Keratin 75 | Covance | 1:100 | NBP1-87845 |
| AE13 | Santa Cruz | 1:100 | sc-57012 |
| AE15 | Santa Cruz | 1:100 | sc-80607 |
| Smooth muscle actin | Abcam | 1:100 | ab5694 |
| CD133 | Abcam | 1:100 | ab19898 |
| Versican | Fisher Sci. | 1:200 | PA1-1748A |
| Ki67 | Abcam | 1:800 | ab15580 |
| Human nucleus | Millipore | 1:100 | MAB1281B |
| Lef-1 | Santa Cruz | 1:100 | sc-8591 |
