# Supplementary material for: Tissue engineering of human hair follicles using a biomimetic developmental approach
Source: Nat Commun. 2018 Dec 13;9:5301. doi: 10.1038/s41467-018-07579-y (PMC6294003; doi:10.1038/s41467-018-07579-y)
Supplement: Supplementary file 2 — Description of Additional Supplementary Files [file 41467_2018_7579_MOESM2_ESM.docx]

**Description of Additional Supplementary Files**

**File Name**: Supplementary Movie 1

**Description**: 3D reconstructed video of K14 positive cells in HSCs with 255 HF per cm^2^
